# Measuring and evaluating standardization of scrub nurse instrument table setups: a multi-center study

**DOI:** 10.1007/s11548-021-02556-1

**Published:** 2022-01-21

**Authors:** Bernhard Glaser, Tobias Schellenberg, Juliane Neumann, Mathias Hofer, Susanne Modemann, Patrick Dubach, Thomas Neumuth

**Affiliations:** 1 Innovation Center Computer Assisted Surgery, Faculty of Medicine, University of Leipzig, Leipzig, Germany; 2 ENT Department, Leipzig University Hospital, Leipzig, Germany; 3 Acqua Klinik, Leipzig, Germany; 4 Department of Otorhinolaryngology, Head and Neck Surgery, Inselspital, University Hospital of Bern, Bern, Switzerland

**Keywords:** Computer-assisted surgery, Intra-operative monitoring, Nurse training, Standardization, Surgical process model, Workflow

## Abstract

****Objectives**:**

In-depth knowledge about surgical processes is a crucial prerequisite for future systems in operating rooms and the advancement of standards and patient safety in surgery. A holistic approach is required, but research in the field of surgical instrument tables, standardized instrument setups and involved personnel, such as nurses, is sparse in general. The goal of this study is to evaluate whether there is an existing standard within clinics for an instrument table setup. We also evaluate to which extent it is known to the personnel and whether it is accepted.

****Materials and Methods**:**

The study makes use of the *Nosco Trainer*, a scrub nurse training and simulation system developed to analyze various aspects of the workplace of scrub nurses. The system contains a virtual instrument table, which is used to perform and record instrument table setups. We introduce a metric which delivers a measurable score for the similarity of surgical instrument table setups. The study is complemented with a questionnaire covering related aspects.

****Results**:**

Fifteen scrub nurses of the Otolaryngology departments at three clinics in Germany and Switzerland performed a table setup for a Functional Endoscopic Sinus Surgery intervention and completed the questionnaire. The analysis of the developed metric with a leave one out cross-validation correctly allocated 14 of the 15 participants to their clinic.

****Discussion**:**

In contrast to the identified similarities of table setups within clinics with the collected data, only a third of the participants confirmed in the questionnaire that there is an existing table setup standard for Functional Endoscopic Sinus Surgery interventions in their facility, but almost three quarters would support a written standard and acknowledge its possible benefits for trainees and new entrants in the operating room.

****Conclusions**:**

The structured analysis of the surgical instrument table using a data-driven metric for comparison is a novel approach to gain deeper knowledge about intra-operative processes. The insights can contribute to patient safety by improving the workflow between surgeon and scrub nurse and also open the way for goal-oriented standardization.

**Supplementary Information:**

The online version supplementary material available at 10.1007/s11548-021-02556-1.

## Introduction

Standardization of surgical interventions with the goal of streamlining processes while improving patient safety is a topic that has been tackled from various angles. Traditional approaches for improving patient safety by reducing ineffective and inefficient surgical processes are the development of surgical guidelines [1] and operating room (OR) standards [2]. These approaches could potentially benefit from novel process analysis methods and implementation strategies. The surgical process itself is nowadays analyzed in-depth with the use of surgical process models [3–5]. Efforts have also been made to improve the efficiency of the OR with modern organizing methods such as Lean Management and Six Sigma techniques [6]. Both analysis and organization are means to get closer to the aspired operating room of the future, which is dependent on the integrity of processes and procedural standards to acquire increased safety, efficacy and cost-effectiveness [7]. By nature, the surgeon has been in the focus of the analysis of surgical interventions for a long time, but the inclusion of all team members and all equipment gains in importance, for example, in team training actions [8] and resource optimization [9]. Crew resource management (CRM) tools such as briefings, debriefings, and checklists are techniques from aviation that are now increasingly being adapted in health care [10–13]. CRM aims to establish a comprehensive culture of safety among teams and uses standardization as a tool for continuous improvement with a focus on details. A popular application of these new influences is the World Health Organization (WHO) Surgical Safety Checklist in the operating room, which includes a review of the instruments available by the nursing team before an incision is made [14, 15]. In addition, professional organizations such as the Association of Perioperative Registered Nurses (AORN) drive standardization efforts by publishing regularly updated guidelines for perioperative practice [16]. Here, the necessity of including a wider range in the efforts for standardization has already been recognized, but detailed research on many influential factors is still missing.

With the introduction of automated surgical assistance and monitoring systems and surgical robotics, all equipment and all team members in the OR need to be taken into account. Most importantly, this includes continuous, automated surveillance of the surgical instruments and the dynamics of human activities in the OR [17].

Research on the topic of surgical instrument tables, standardized instrument setups and involved personnel such as nurses is, however, sparse in general. On the topic of the prevention of retained instruments, there is extended analysis available on possible aspects that can trigger miscounts, such as distractions, but the organization of the instrument table itself is not taken into account [18, 19]. Fort and Fitzgerald used simulation as a training tool to improve perioperative nursing outside of a real clinical setting [20]. Here, they incorporated the setup of the instrument table as a task of the curriculum. Gerbrands et al. have analyzed the work area of the scrub nurse from an ergonomic point of view and suggested improvable aspects, including an ergonomically designed instrument table [21]. Pérez-Vidal et al. have touched on the topic of instrument table organization when describing the development of a robotic scrub nurse [22].

On the topic of the surgical instrument table for Functional Endoscopic Sinus Surgery (FESS), Schmitz et al. have improved the changeover times during FESS with a modified instrument table [23], for which the placement of the three most commonly used instruments is performed by the surgeon himself in a standardized and uniform manner.

As part of our broader research strategy to address procedural standards in the OR with a focus on the scrub nurse, we have both presented both a system for intra-operative identification of surgical instrument movements on the instrument table [25] and an interactive training system for scrub nurses [26]. We have also addressed the topic of terminological standards and the use of varying nomenclature for surgical instruments in FESS by scrub nurses [27].

In this follow-up study, we use the newly gained possibilities of a simulated instrument table to analyze the setup of the surgical instrument table. We aim to evaluate whether there is an existing standard within clinics for the setup of an instrument table based only on the data-driven analysis of performed table setups. We also evaluate to which extent it is known to the personnel and whether it is accepted, with implications for standardization efforts.

## Methods

This paragraph will first introduce the structural system setup of the system used in the study, followed by the introduction of the developed similarity metric for instrument tables.

### System design: structural system setup

**Fig. 1:**
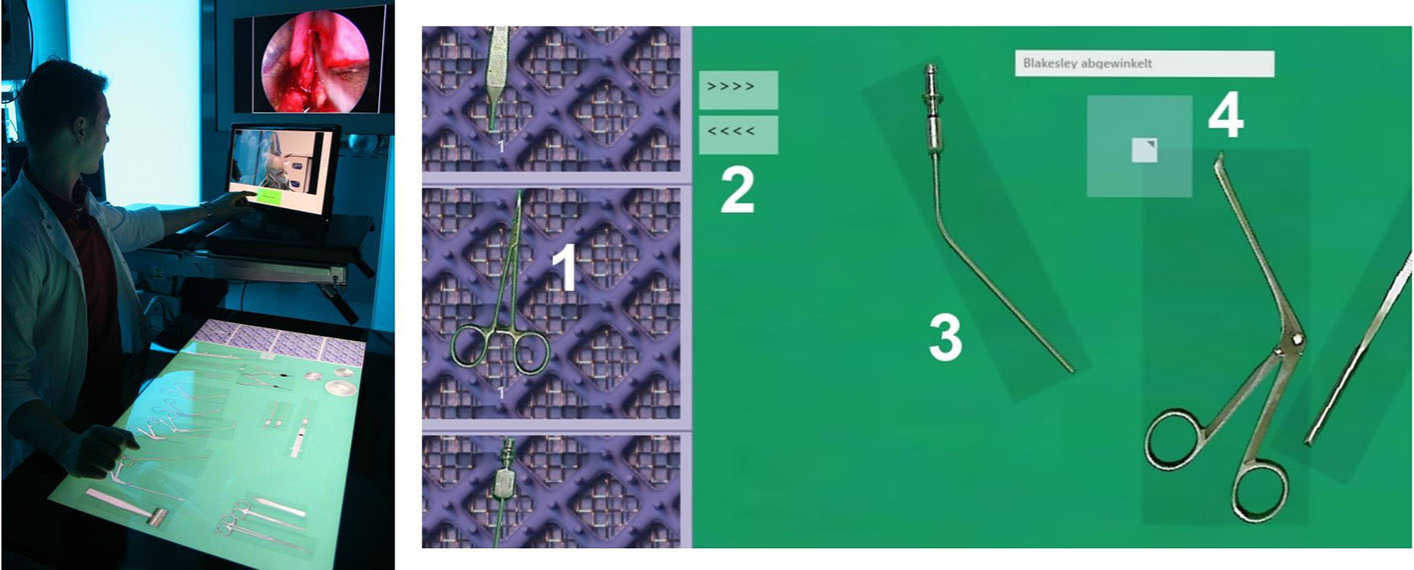
Left: Setup of the trainer system at the demonstration OR with the *Microsoft PixelSense* system used to simulate the instrument table. Right: Magnified GUI detail with main features of the instrument table simulation software numbered from one to four

The study setup is based on the *Nosco Trainer* system, which was previously developed [26] at the ICCAS, Leipzig University (Germany) and is undergoing evaluation to address various aspects of the scrub nurse workplace [27]. Figure 1 (left) shows a demonstration setup of the trainer system and the interactive table used to simulate the instrument table.

With this system, a participant can perform instrumentation for surgical interventions virtually by arranging and selecting instruments on a *Microsoft* PixelSense (formerly known as *Microsoft Surface*) interactive surface computing platform [28], hereafter referred to as the *instrument table system*. The simulation is supported by a touch screen which displays the surgical activities and allows interaction with the surgeon. Figure 1 (right) shows a magnified graphical user interface (GUI) detail of the instrument table system with the main features of the instrument table simulation software. The instrument tray container is simulated using a list displaying slightly reduced images of all instruments (1). The background of the list is an image of an original surgical tray mat. The list can be scrolled with drag and drop finger movements, similar to scrolling on a smartphone. If an instrument is selected in the tray, it can be placed on the table by pushing a special button (2). All instruments on the table are surrounded by a semitransparent gray bounding box (3), which indicates the area in which the instrument can be touched for interaction. Instruments can be moved, turned 360 degrees, and stacked. If an instrument is touched, a small white square button appears on its upper left corner for several seconds (4). If this button is pressed, a label with the instrument name is shown.

### System design: table similarity metric

A metric was developed to introduce a measurable score for the similarity of two surgical instrument tables. Let $s_\mathrm{tab}(A,B)$ be the normalized sum of all individual scores $s_\mathrm{inst}(I_x)$ for each instrument $I_x$ of the two tables *A* and *B* defined asDepending on whether the instrument $I_x$ is available on both tables or not, the individual score $s_\mathrm{inst}(I_x)$ for each instrument varies with

$$\begin{aligned} s_\mathrm{inst}(I_x) = {\left\{ \begin{array}{ll} w_\mathrm{trans} * d_\mathrm{trans}(I_x) + w_\mathrm{rot} * d_\mathrm{rot}(I_x) &{} I_x\in I^{(A)} \wedge I_x\in I^{(B)} \\ w_\mathrm{miss} &{} {\text {otherwise}} \end{array}\right. } \end{aligned}$$

using $w_\mathrm{trans}$, $w_\mathrm{rot}$ and $w_\mathrm{miss}$ as weighting variables, which allows configuring the influence of translational and rotational differences as well as the influence of missing instruments. The individual score for an instrument $I_x$ is composed using the translational information with

$$\begin{aligned}&d_\mathrm{trans}(I_x) \\&= \sqrt{(xpos(I^{(A)}_x)-xpos(I^{(B)}_x))^2 + (ypos(I^{(A)}_x)-ypos(I^{(B)}_x))^2} \end{aligned}$$

as the Euclidean distance between the instruments with

$$\begin{aligned} 0 \le xpos(I^{(Z)}_x) \le 1920 \quad {\hbox {and}} \quad 0 \le ypos(I^{(Z)}_x)) \le 1080 \end{aligned}$$

defining the position of instrument $I_x$ on the x- and y-axis on the respective table. It is amended by the rotational deviation represented as the minimal angle needed to transform one instrument angle into the other, defined as

$$\begin{aligned} d_\mathrm{angle}(I_x) = |{\hbox {angle}}(I^{(A)}_x) - {\hbox {angle}}(I^{(B)}_x)| \quad {\hbox {and}}\\ \quad d_\mathrm{rot}(I_x) = \min \Big ( d_\mathrm{angle}(I_x), 360 - d_\mathrm{angle}(I_x)\Big ). \end{aligned}$$

By definition, the metric is a symmetrical function with

$$\begin{aligned} s_\mathrm{tab}(A,B) = s_\mathrm{tab}(B,A). \end{aligned}$$

Higher values of $s_\mathrm{tab}$ indicate a decreased similarity between the involved tables, whereas a value of 0 indicates identical table setups.

**Fig. 2:**
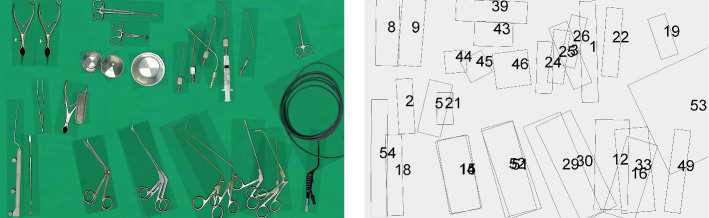
Screenshot for an example instrument table of the Acqua Klinik (*acqua4*) and the associated schematic information. A legend is provided in supplementary material [29]. The chosen table is the most similar to all other tables of the study according to Table 1

## Evaluation study

### Evaluation study design

A multi-center study was conducted to analyze similarities and differences as well as certain backgrounds of the instrument table setup of scrub nurses under laboratory conditions. Participants were recruited for the study by the ENT physicians, who also supervised the medical aspects. The study was conducted in three different hospitals in order to include a multifaceted target audience and to acquire a significant group of participants. The ENT departments of three different hospitals participated, including the Acqua Klinik Leipzig, Germany, the Inselspital Bern, Switzerland and the University Medical Center Leipzig, Germany. Each clinic provided a secluded room for demonstrator setup during the period of the study. A typical ENT intervention called Functional Endoscopic Sinus Surgery (FESS) was selected as the basis for the study. For this purpose, a complete surgical tray was photographed and digitized with a surgical tray management software presented in a previous article [25]. The tray shown was the one which is used in the Acqua Klinik for this type of intervention. A detailed list of all instruments appearing on table setups in the study is provided in supplementary material [29]. Five participants of each clinic were interviewed, with a total sum of fifteen participants. In the first part of the study, the participants were asked to perform the initial surgical instrument table setup using the instrument table system. There was no time limit for the participants. During post-processing, each table setup was compared with each of the gathered table setups using the presented table similarity metric. $w_\mathrm{trans}=1$ and $w_\mathrm{rot}=1$ were set constant, and $w_\mathrm{miss}$ was varied. Additionally, a leave one out cross-validation was performed in order to evaluate, whether it is possible to classify the clinic affiliation of an instrument table by comparing it to all the other tables. Therefore, in each case one table was removed, and with the remaining 14 tables, the average similarity values were determined for each clinic. The lowest average similarity value for a clinic was considered to be the classification result in this very basic comparator. Finally, to determine the table most similar to all other tables according to the metric, the total average was calculated as well for each individual table. In the second part of the study, participants were asked to answer a series of questions during an interview as a supplement to the first part.

### Evaluation study results

Ten of the 15 participants were female, 5 were male. Three of the participants were between 18 and 30 years, six between 31 and 40 years, five between 41 and 50 years and one between 51 and 60 years. Thirteen of 15 participants spoke German natively. All participants were fluent in German. All of the participants were smartphone users. All participants had received a classic nursing education with a continuing education as OR specialist with the exception of three participants in the Swiss hospital, who had received a training called degreed operating room technician^1^ or technical operation assistant^2^, respectively. The 15 participants had an average of $15.7 \pm 9.4$ years of experience as nurses, of these, an average of $12.8 \pm 9.8$ years were as scrub nurses. Eight of the 15 participants were trained in the same OR in which they were working during the interview. All of the participants had performed surgical instrumentation for a FESS before and were generally familiar with the type of intervention. Figure 2 shows an example instrument table and the associated schematic information.

Table 1 shows the leave one out cross-validation results with $s_\mathrm{tab}(A,B)$ for all table combinations (weighting parameters $w_\mathrm{trans}=1$, $w_\mathrm{rot}=1$ and $w_\mathrm{miss}=1000$). Table 2 presents the results for the first group of questions, in which participants were asked about instrument table setup standards (Q1.1–Q5). Table 3 presents the results for a group of questions for which participants were asked to confirm or decline possible influences on their initial table setup. (Q6.1–Q6.5). A data set with the raw data of the instrument table setups in XML and corresponding images is provided online [29].

**Table 1:** Leave one out cross-validation results with $s_\mathrm{tab}(A,B)$ for all table combinations (weighting parameters $w_\mathrm{trans}=1$, $w_\mathrm{rot}=1$ and $w_\mathrm{miss}=1000$)

	insel $\varnothing$	acqua $\varnothing$	ukl $\varnothing$	total $\varnothing$
insel1	**784.8**	897.4	946.1	882.6
insel2	861.3	*783.1*	876.0	838.6
insel3	**824.6**	907.5	852.6	864.2
insel4	**812.3**	844.0	923.5	863.3
insel5	**755.5**	803.0	883.4	818.1
acqua1	861.0	**597.2**	804.2	765.4
acqua2	839.5	**575.2**	823.7	758.3
acqua3	828.3	**591.2**	822.6	758.5
acqua4	803.5	**571.5**	845.9	***752.4***
acqua5	902.7	**678.1**	801.0	802.2
ukl1	902.8	783.9	**739.3**	813.6
ukl2	892.6	810.1	**776.4**	830.0
ukl3	905.5	851.2	**778.1**	849.7
ukl4	868.8	823.8	**779.1**	827.1
ukl5	912.0	828.4	**714.8**	825.8

Correct classifications are marked in bold, incorrect classifications are marked in italics. Bolditalics indicates the most similar table to all other tables of the study. A detailed comparison of each single table is provided in supplementary material [29]

**Table 2:** Closed questions covering instrument table setup standards (possible answers include *yes* and *no*), percent of agreement per clinic and in total

Question	insel %	acqua %	ukl %	Total %
Is there a GENERAL standard for how to set up an instrument table in your team, e.g., that a scalpel has to lie with its sharp side away from the scrubbing person? (Q1.1)	40.0	60.0	60.0	53.3
Q1.1 applies & Do you know who made this standard? (Q1.2)	40.0	20.0	20.0	26.7
Q1.1 applies & Q1.2 applies & Is this standard written down somewhere? (Q1.3)	40.0	20.0	20.0	26.7
Q1.1 applies & Q1.2 applies & Q1.3 applies & Do you have access to this standard? (Q1.4)	40.0	0.0	20.0	20.0
Is there a SPECIAL standard for how to set up an instrument table for a FESS intervention in your team? (Q2.1)	40.0	20.0	40.0	33.3
Q2.1 applies & Do you know who made this standard? (Q2.2)	20.0	20.0	40.0	26.7
Q2.1 applies & Q2.2 applies & Is this standard written down somewhere? (Q2.3)	20.0	0.0	40.0	20.0
Q2.1 applies & Q2.2 applies & Q2.3 applies & Do you have access to this standard? (Q2.4)	20.0	0.0	40.0	20.0
Do you know any literature which describes how a table should be set up for an operation? (Q3)	40.0	20.0	80.0	46.7
Would you support a written down standard per operation type for the setup of the instrument table? (Q4)	80.0	40.0	100.0	73.3
Could a written down standard per operation for the setup of the instrument table help trainees and new entrants in the OR to familiarize themselves quicker? (Q5)	80.0	40.0	100.0	73.3

**Table 3:** Answers to superordinate closed question “My initial table setup is influenced by the following factors:” (possible answers include yes and no), percent of agreement per clinic and in total

Influencing factor	insel%	acqua %	ukl %	Total %
The surgeon: There are surgeons for which I set up the table differently than for their colleagues at the same intervention type. (Q6.1)	20.0	20.0	60.0	33.3
By chance/Daily mood: It happens that I set up the table for an intervention of the same type differently than I did the last time. (Q6.2)	0.0	0.0	0.0	0.0
My personal preference: I set up the table as I consider it ideal. (Q6.3)	80.0	100.0	100.0	93.3
My training: I set up the table in the way I was trained. (Q6.4)	60.0	60.0	80.0	66.7
In-house standard: I set up the table as dictated by the in-house standard. (Q6.5)	60.0	20.0	20.0	33.3

## Discussion

As a basis for this study, we created a system to simulate an instrument table with the associated surgical instrument tray as part of a scrub nurse training system [26]. In this paper we developed a metric to determine the level of similarity between surgical instrument tables based on the position, orientation as well as presence and absence of common instruments. A study was conducted to evaluate the level of standardization for instrument table setups of scrub nurses from three different clinics and to investigate related influence factors. The results indicate that the working environment of the OR influences the way that the initial table is arranged.

As shown in Table 1, all of the tables of the study with the exception of one table (*insel2*) are allocated to the correct associated clinic using the presented instrument table similarity metric. This equals a very good rate of correct classifications with 93.3%. Although the results of the table setup comparison clearly indicate a certain kind of standardization of the table setup within each clinic, the results of the additional interview questions presented in Table 2 show that the existence of such a standard is not generally acknowledged. Only 53.3% of the participants confirmed a general standard for how to set up an instrument table in the team (Q1.1), compared to only 33.3% that confirmed the existence of a standard for the setup of an instrument table in the team for the chosen FESS intervention (Q2.1). The questions covering knowledge about the creators of the standards (Q1.2, Q2.2) and the access to them (Q1.3, Q2.3, Q1.4, Q2.4) strengthen the implication that a formal standard is not anchored amongst the team members. The fact that less than half of the participants stated that they were not aware of any literature covering the topic of instrument table setups (Q4) also supports the impression that little attention is given to the topic during formal training. The topic of how to set up the instrument table for an intervention is typically taught on the job by a senior scrub nurse supervising the newcomers. 66.7% stated that they set up the table as they were trained for it (Q6.4), compared to 33.3% who stated they were following an in-house standard (Q6.5). Sometimes, this can also be influenced by additional factors. For example, one third of the participants mentioned that there are surgeons for whom they set up the instrument table differently (Q6.1). In general, however, 93.3% stated that they set up the table in a way they personally consider ideal (Q6.3). Interestingly, a vast majority (73.3% percent) of the participants agreed that they would support a written down standard for each operation type for instrument table setup (Q4), and that this could help trainees and new entrants in the OR to familiarize themselves more quickly (Q5).

## Conclusions

The structured analysis of the surgical instrument table is a novel approach to gain deeper knowledge about intra-operative processes. It can provide new insights for improving the workflow between surgeon and scrub nurse and open the way for goal-oriented standardization. This contributes to less disruptions in the surgical flow and therefore the prevention of surgical errors [24]. To the best of our knowledge, the presented article is the first comparative analysis of surgical instrument table setups. The involved simulation system allows gathering precise location data of the instruments involved. It is independent from intervention type and easily transferable to different surgical disciplines. It can also be transferred to a real instrument table by using the 2D camera input of a surveilled table [25] and slight modifications to the presented metric input data, which is based on screen coordinates. The results of the leave-one-out cross-validation with a 93.3% percentage of correct attributions of a single table to its associated clinic clearly expose an existing standard for the instrument table setup within each participating clinic. However, the interview results lead to the assumption that it is less influenced by written and known guidelines and more by acquired habits that develop within teams working in the same environment. A vast majority (73.3%) of the participants, however, supports the concept of a written standard for each operation type for the setup of the instrument table, confirming possible benefits for a better novice OR personnel training. Standards for the instrument table could also contribute in the everyday work of experienced scrub nurses, for example, for quicker orientation if a table is taken over from another person during long interventions. This becomes even more important when considering that personnel in other surgical disciplines than ENT often work with multiple tables and large amounts of similar-looking instruments. Here, not only the continuity of the surgical flow could be supported, but this could also enhance safety. During interventions for which certain instruments are exposed to tumor tissue, for instance, contaminated instruments must not come in contact with healthy tissue at later stages. As a general rule, these instruments are not marked, but a possible backup scrub nurse is informed verbally about the location of these instruments on the table. A standard could contribute to patient safety here. Follow-up projects will extend the work to other intervention types and surgical domains and also transfer the results into concepts for scrub nurse training programs.

## Supplementary Information

Below is the link to the electronic supplementary material.

Supplementary material 1 (zip 11257 KB)

## Footnotes

1 In German: Dipl. Fachfrau/-mann Operationstechnik.

2 In German: technische(r) Operationsassistent(in) (TOA).
