# Supplementary figures and images for: Measuring and evaluating standardization of scrub nurse instrument table setups: a multi-center study
Source: Int J Comput Assist Radiol Surg. 2022 Jan 21;17(3):479–85. doi: 10.1007/s11548-021-02556-1 (PMC8873066; doi:10.1007/s11548-021-02556-1)

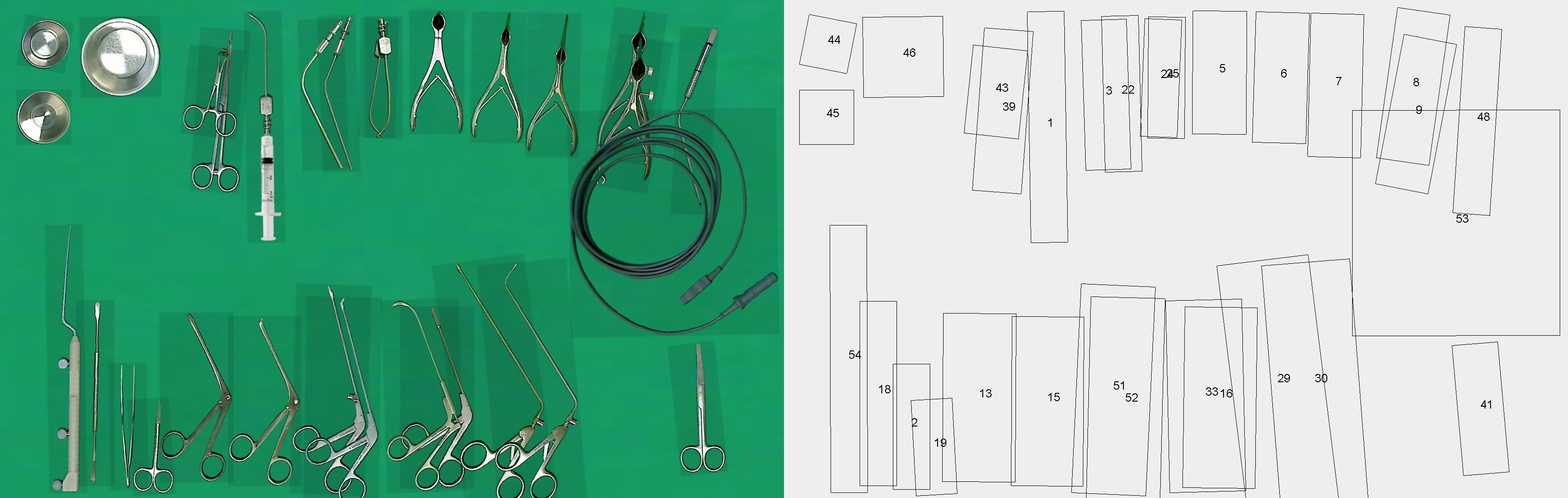

Supplement: Supplementary file 1 — Supplementary material 1 (zip 11257 KB) [file 11548_2021_2556_MOESM1_ESM.zip › instrument-table-dataset-master/jpg/acqua1.jpg]

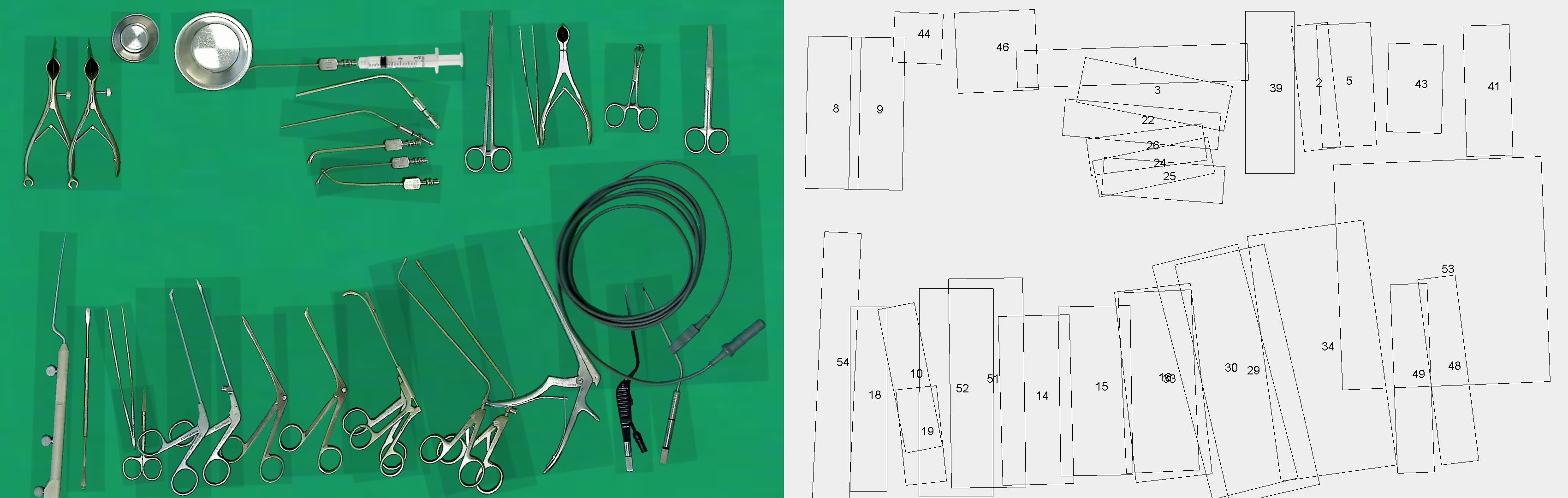

Supplement: Supplementary file 1 — Supplementary material 1 (zip 11257 KB) [file 11548_2021_2556_MOESM1_ESM.zip › instrument-table-dataset-master/jpg/acqua2.jpg]

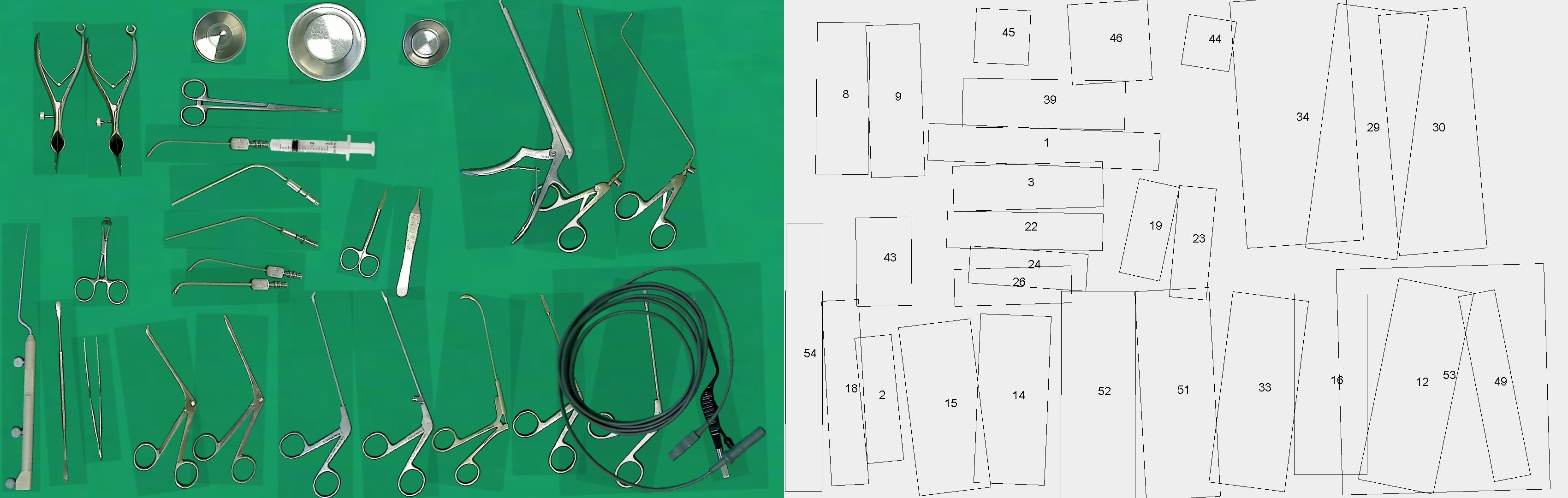

Supplement: Supplementary file 1 — Supplementary material 1 (zip 11257 KB) [file 11548_2021_2556_MOESM1_ESM.zip › instrument-table-dataset-master/jpg/acqua3.jpg]

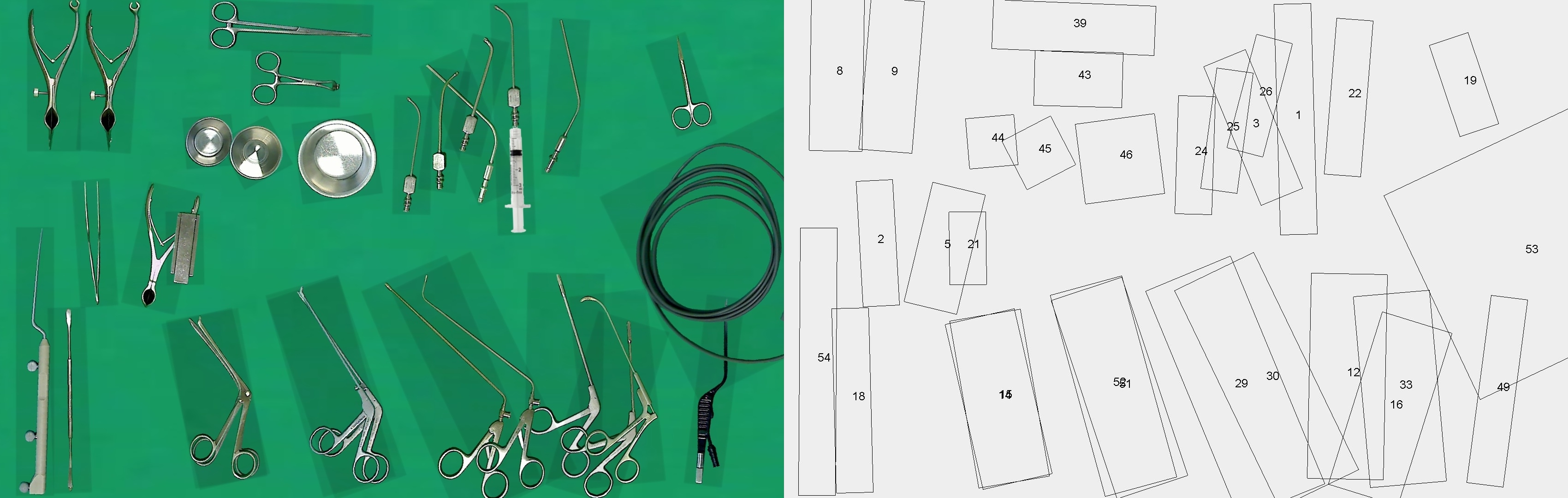

Supplement: Supplementary file 1 — Supplementary material 1 (zip 11257 KB) [file 11548_2021_2556_MOESM1_ESM.zip › instrument-table-dataset-master/jpg/acqua4.jpg]

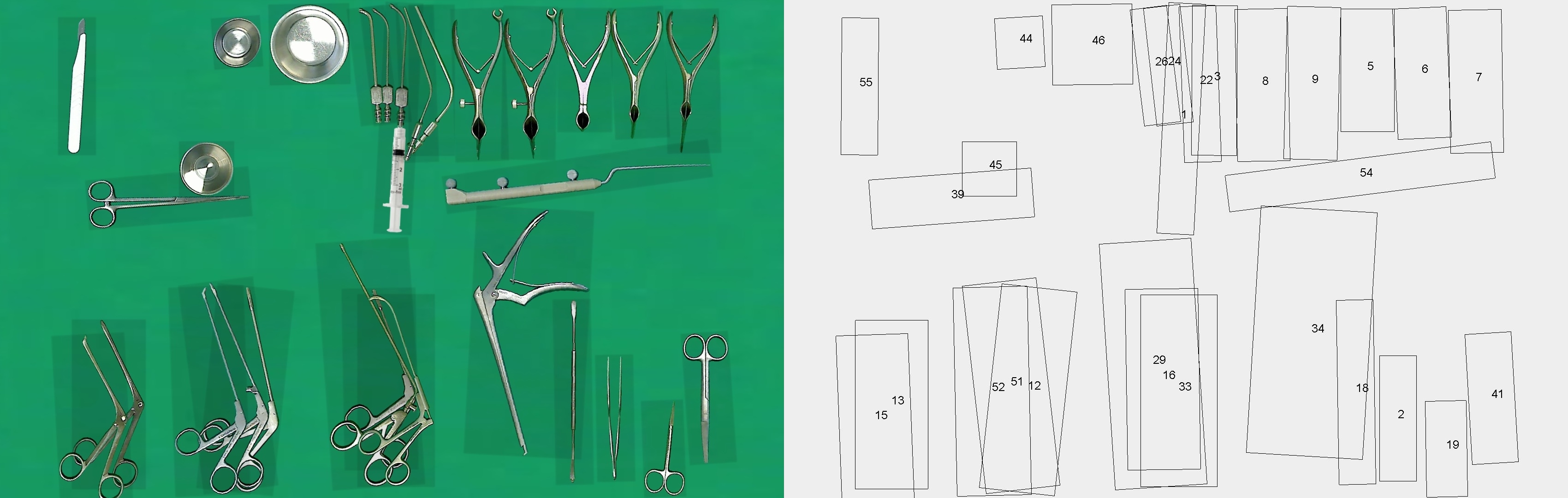

Supplement: Supplementary file 1 — Supplementary material 1 (zip 11257 KB) [file 11548_2021_2556_MOESM1_ESM.zip › instrument-table-dataset-master/jpg/acqua5.jpg]

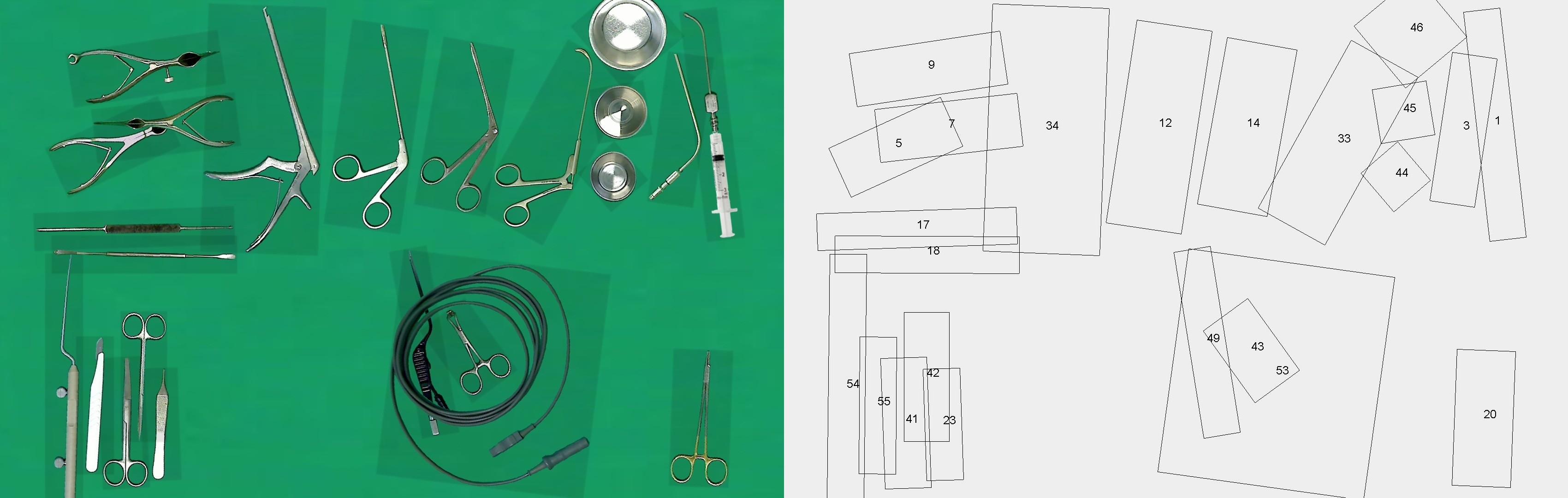

Supplement: Supplementary file 1 — Supplementary material 1 (zip 11257 KB) [file 11548_2021_2556_MOESM1_ESM.zip › instrument-table-dataset-master/jpg/insel1.jpg]

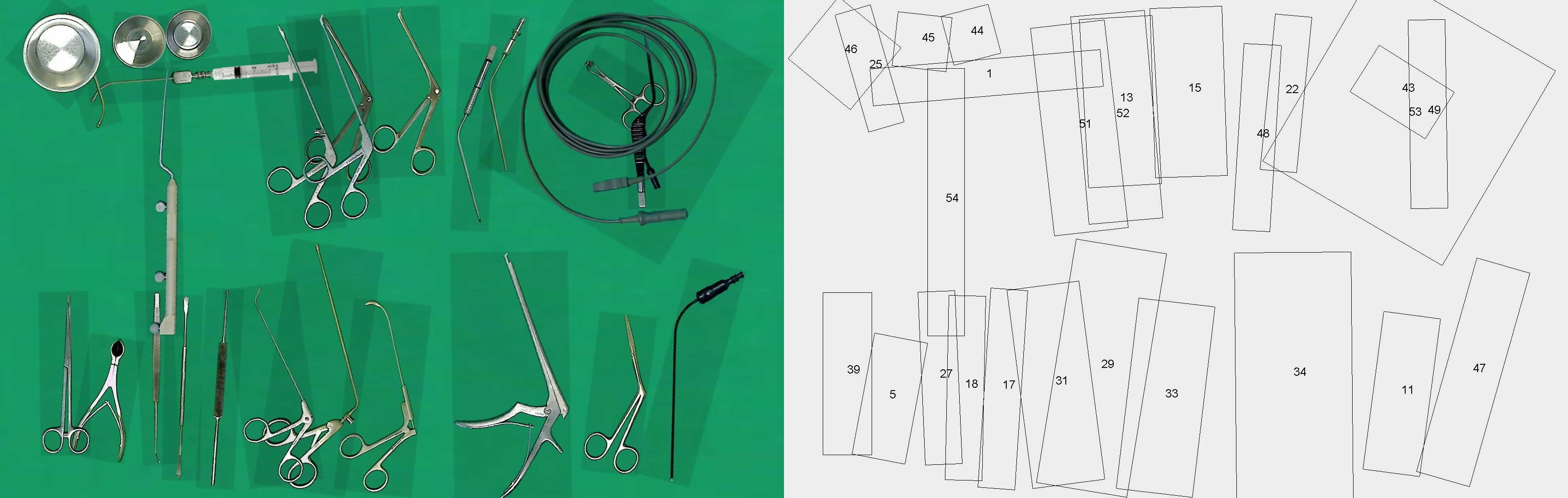

Supplement: Supplementary file 1 — Supplementary material 1 (zip 11257 KB) [file 11548_2021_2556_MOESM1_ESM.zip › instrument-table-dataset-master/jpg/insel2.jpg]

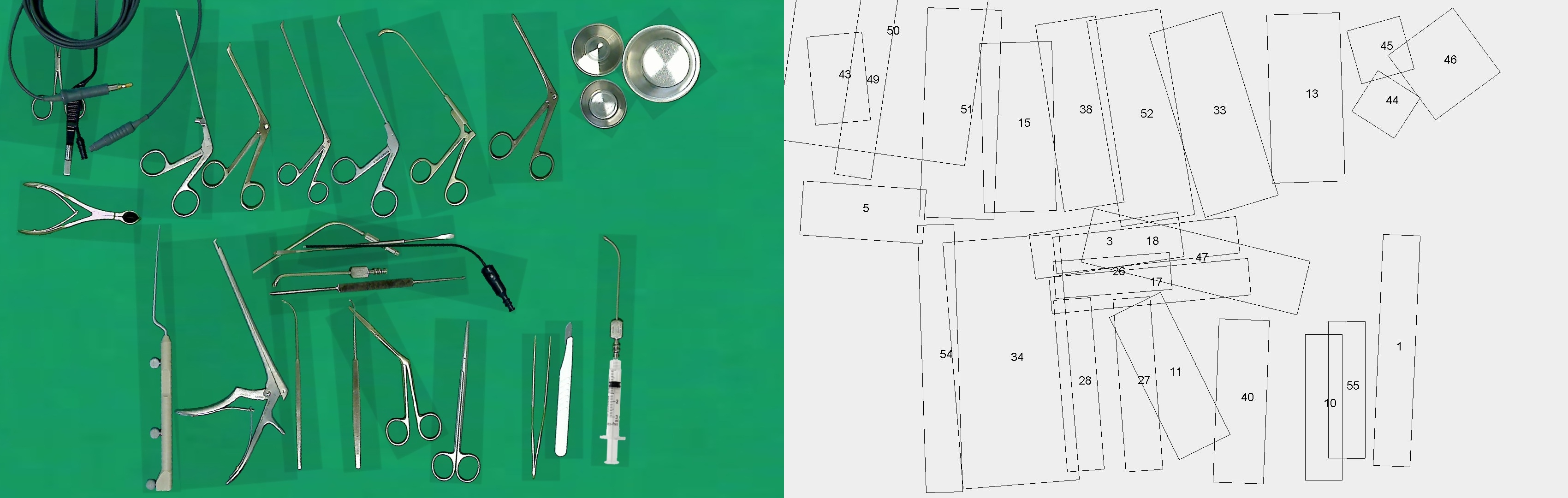

Supplement: Supplementary file 1 — Supplementary material 1 (zip 11257 KB) [file 11548_2021_2556_MOESM1_ESM.zip › instrument-table-dataset-master/jpg/insel3.jpg]

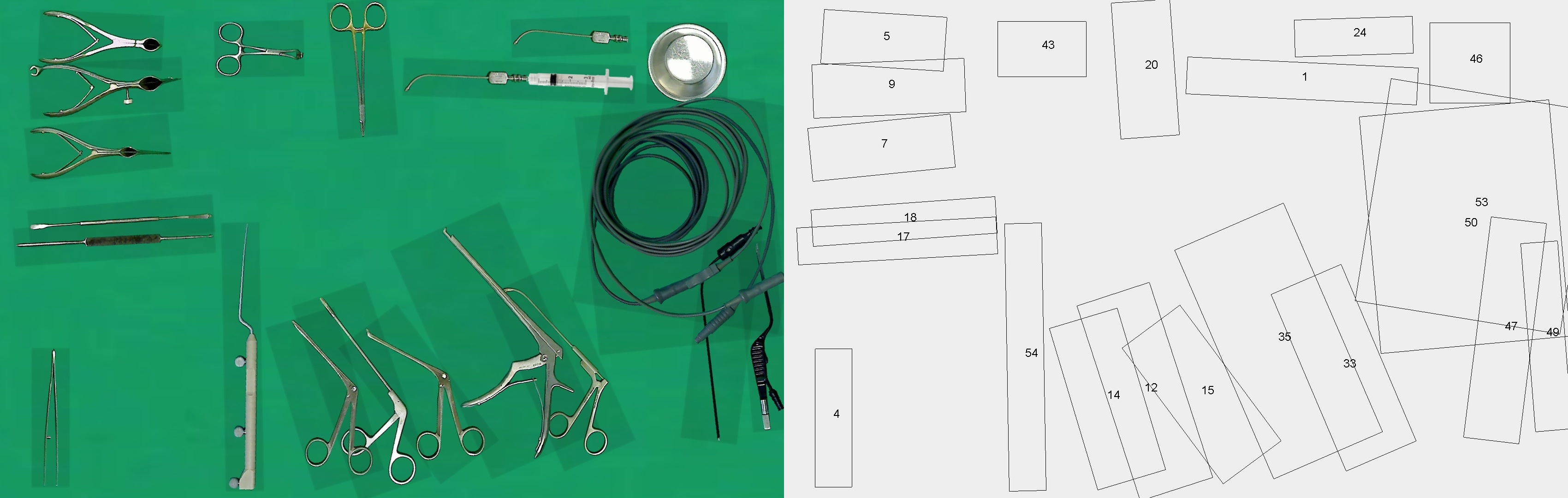

Supplement: Supplementary file 1 — Supplementary material 1 (zip 11257 KB) [file 11548_2021_2556_MOESM1_ESM.zip › instrument-table-dataset-master/jpg/insel4.jpg]

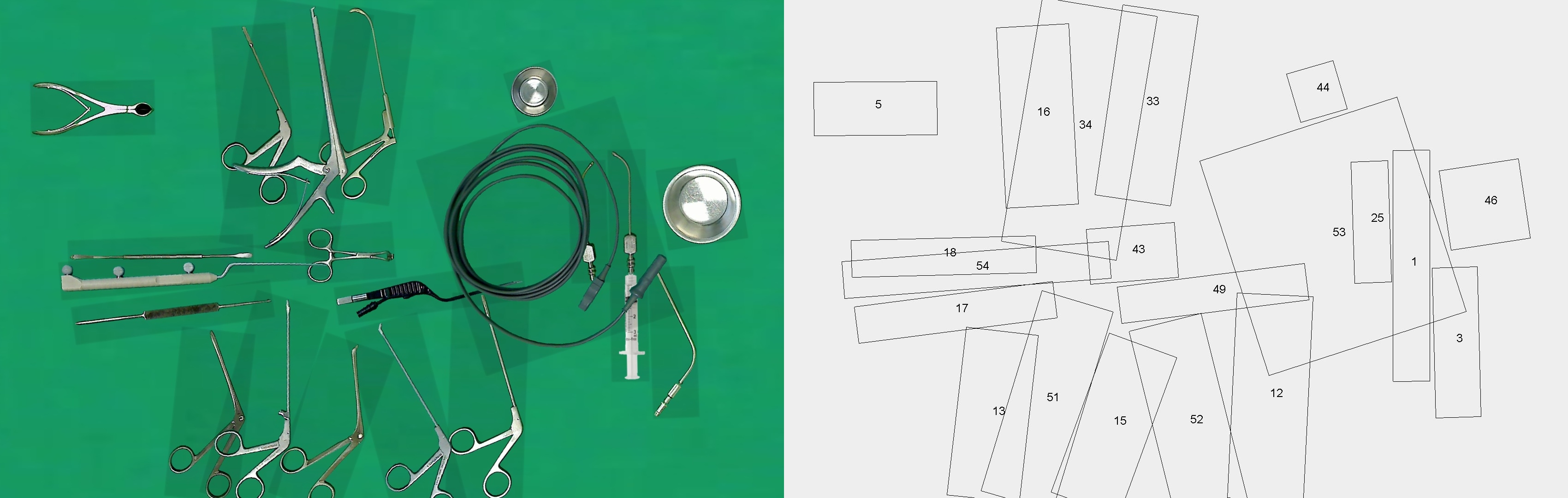

Supplement: Supplementary file 1 — Supplementary material 1 (zip 11257 KB) [file 11548_2021_2556_MOESM1_ESM.zip › instrument-table-dataset-master/jpg/insel5.jpg]

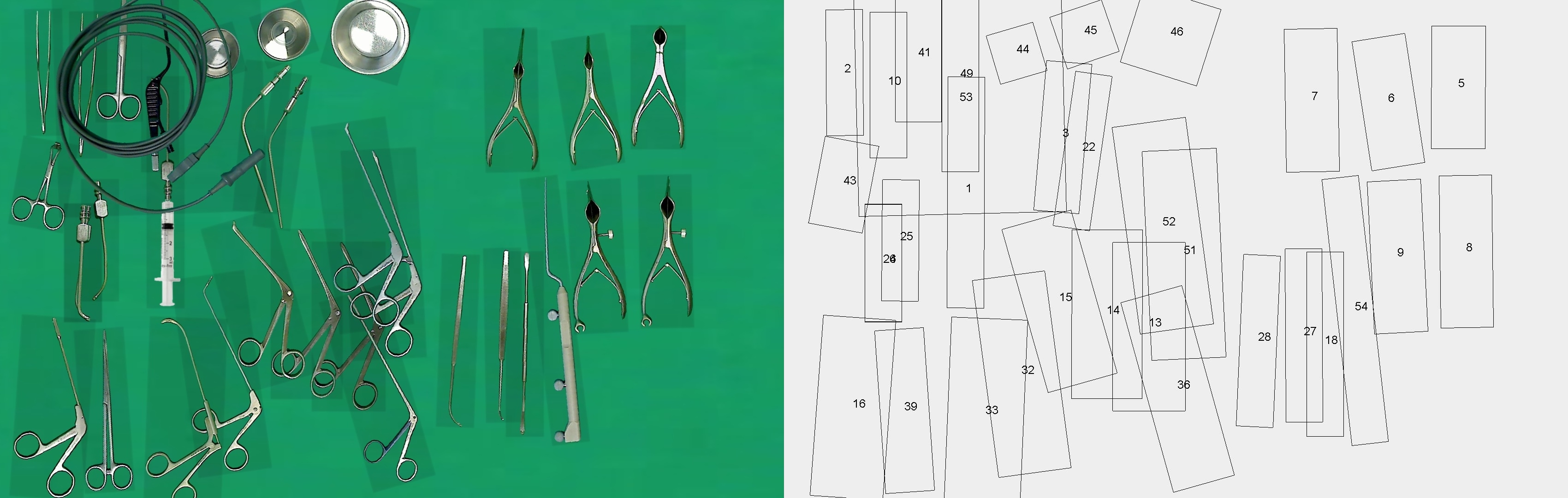

Supplement: Supplementary file 1 — Supplementary material 1 (zip 11257 KB) [file 11548_2021_2556_MOESM1_ESM.zip › instrument-table-dataset-master/jpg/ukl1.jpg]

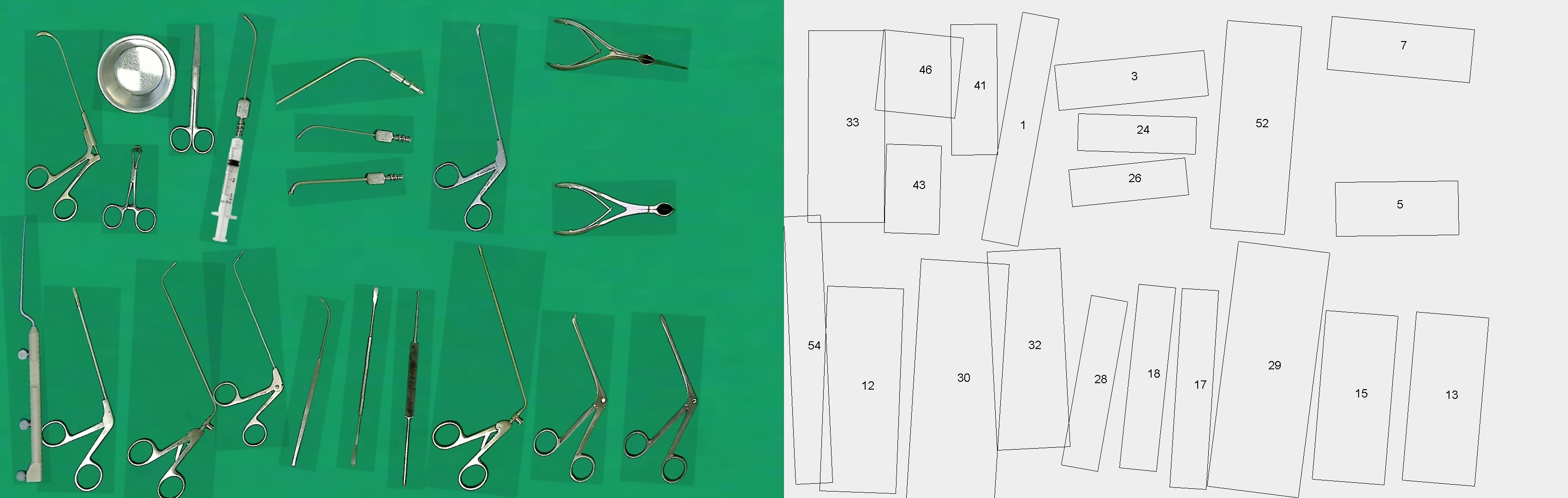

Supplement: Supplementary file 1 — Supplementary material 1 (zip 11257 KB) [file 11548_2021_2556_MOESM1_ESM.zip › instrument-table-dataset-master/jpg/ukl2.jpg]

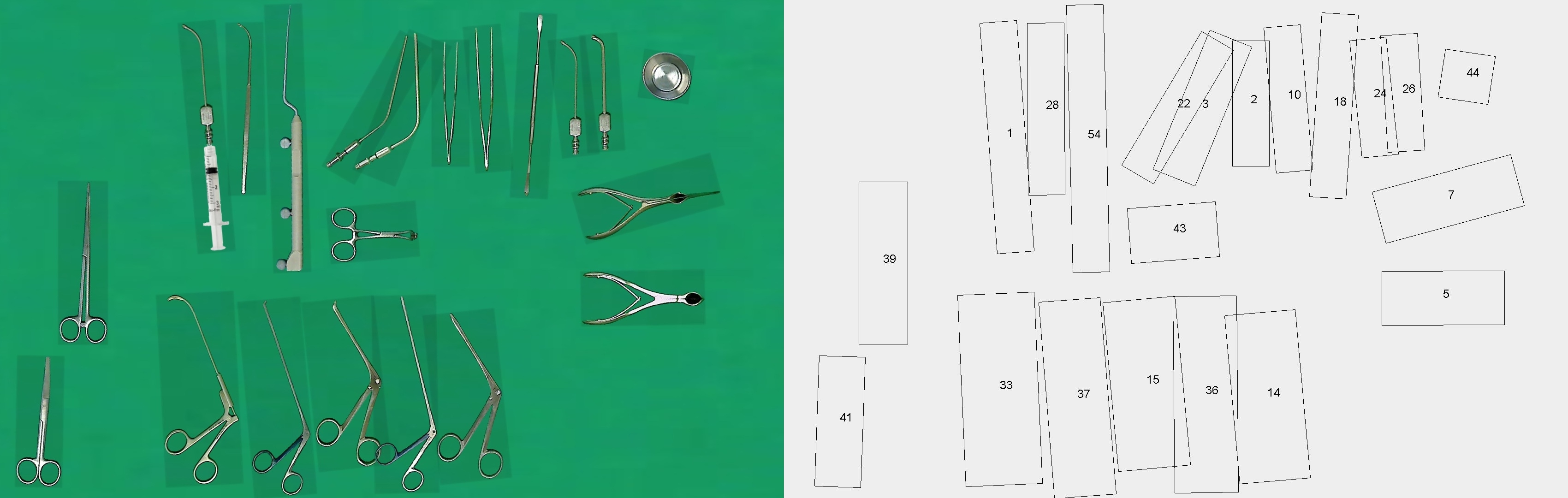

Supplement: Supplementary file 1 — Supplementary material 1 (zip 11257 KB) [file 11548_2021_2556_MOESM1_ESM.zip › instrument-table-dataset-master/jpg/ukl3.jpg]

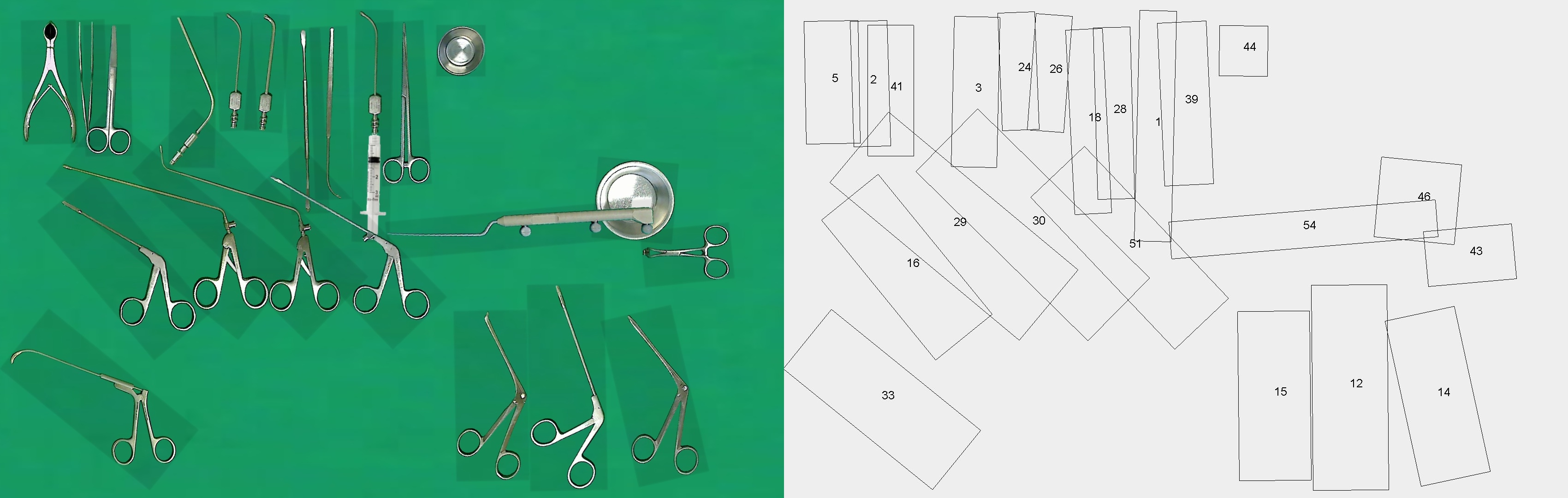

Supplement: Supplementary file 1 — Supplementary material 1 (zip 11257 KB) [file 11548_2021_2556_MOESM1_ESM.zip › instrument-table-dataset-master/jpg/ukl4.jpg]

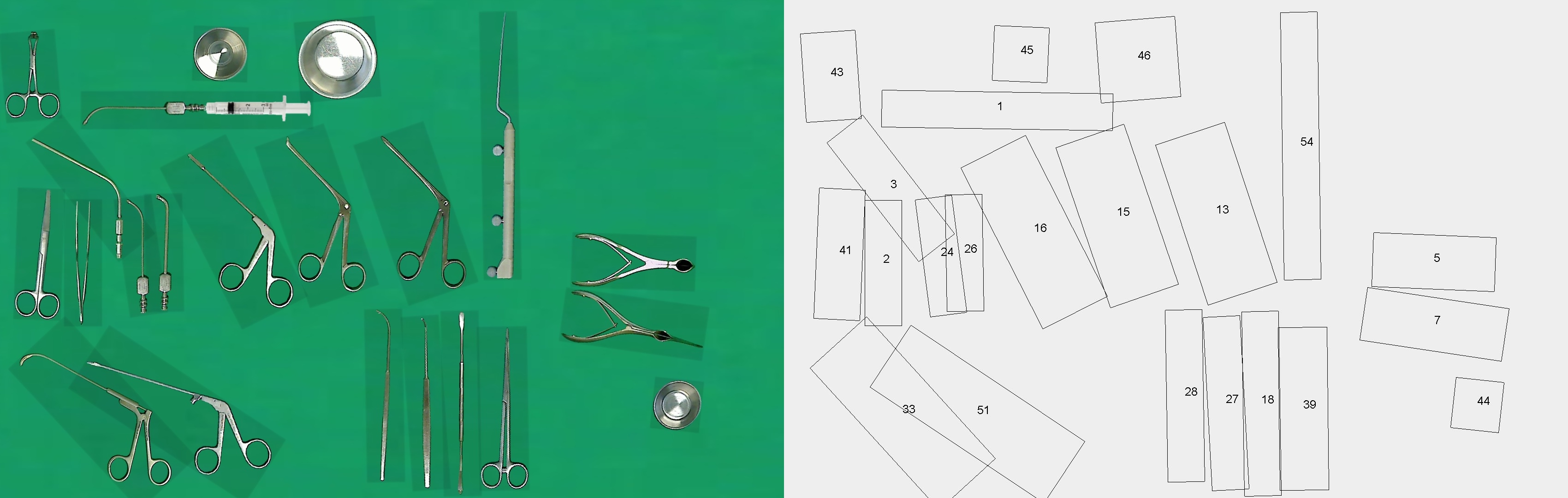

Supplement: Supplementary file 1 — Supplementary material 1 (zip 11257 KB) [file 11548_2021_2556_MOESM1_ESM.zip › instrument-table-dataset-master/jpg/ukl5.jpg]
